# MicroRNAs in Development

**DOI:** 10.1100/tsw.2006.313

**Published:** 2006-03-26

**Authors:** Danielle Maatouk, Brian D. Harfe

**Affiliations:** University of Florida College of Medicine, Department of Molecular Genetics and Microbiology, Gainesville, FL 32610, USA

**Keywords:** Dicer, microRNAs, mice, RISC, RNase III

## Abstract

Over 10 years ago, the lab of Victor Ambros cloned an unusual gene, *lin-4*, which encodes two small RNA transcripts[1]. In the past few years, hundreds more of these tiny transcripts, termed microRNAs (miRNAs), have been uncovered in over a dozen species. The functions of the first two miRNAs, *lin-4* and *let-7*, were relatively easy to identify since they were found in forward genetic screens in Caenorhabditis elegans[1,2,3]. However, uncovering the functions of the growing list of miRNAs presents a challenge to developmental biologists. This review will describe our current understanding of how miRNAs regulate gene expression and will focus on the roles these noncoding RNAs play during the development of both invertebrate and vertebrate species.

## INTRODUCTION

### MicroRNA Transcription and Processing

MicroRNAs (miRNAs) are the newest members of the noncoding class of RNAs. The majority of miRNAs are located in intergenic regions and are transcribed from their own promoters, while a subset of miRNAs are located within the introns of protein coding genes[4,5,6]. Many of these are found in the sense orientation suggesting that they may be processed from the pre-mRNA transcript[7,8,9]. For example, in *Drosophila*, *miR-11* is located within an intron of the *E2f* gene and both genes have overlapping expression patterns[7]. Similarly, miRNAs can be found in clusters that are expressed as polycistronic transcripts, with each miRNA being subsequently processed out of the primary transcript[5,6]. While half of the miRNAs identified in *D. melanogaster* are located in clusters[7], the majority of miRNAs in *Caenorhabditis elegans* and humans are not clustered and are located in intergenic regions[9,10].

The initial miRNA transcript forms a large hairpin structure[11,12]. This structure, termed the primary-miRNA transcript (pri-mRNA), is processed by the Microprocessor complex that is composed of the RNase III endonuclease Drosha, and the double-stranded-RNA-binding protein DGCR8 (in humans) or Pasha (in *Drosophila* and *C. elegans*)[13,14]. Within the nucleus, this complex cleaves the pri-miRNA at the base of the stem-loop, releasing the premature miRNA (pre-miRNA), a 60–70 nt stem-loop RNA molecule. The pre-miRNA is then actively transported to the cytoplasm via Exportin-5[15,16] where it is further processed by Dicer, another RNase III endonuclease[14]. Dicer cleaves the pre-miRNA approximately two helical turns from the site of Drosha cleavage, releasing the mature miRNA duplex of about 22 nt[17,18,19].

Once the miRNA duplex has formed, one strand is incorporated into the multiprotein RNA-Induced Silencing Complex (RISC). RISC is composed of a number of proteins, including Argonaute, and facilitates the association of the miRNA with its target genes (reviewed in [20]). While rarely found in animals, perfect complimentarity between the miRNA and its target sequence results in Argonaute-mediated cleavage of the target transcript between nucleotides 10–11 of the miRNA[21,22]. More commonly, imperfect pairing between the miRNA and its target results in inhibition of gene translation[2,23] and sequestration of miRNA-bound transcripts in cytoplasmic P-bodies, which are sites of mRNA degradation[24]. However, recent work by Bagga et al.[25] has clearly shown that two miRNAs (*lin-4* and *let-7*) thought to function by inhibiting protein synthesis instead cause the degradation of their target transcripts. Interestingly, unlike the RISC-mediated endonuclease cleavage that occurs on perfect miRNA-target binding, Bagga et al. did not detect cleavage products, suggesting that target mRNA degradation may occur by a 5'-3' exonuclease-dependent mechanism. The colocalization of RISC components with cytoplasmic P-bodies supports this model[26]. Additionally, disruption of P-bodies has recently been shown to cause defects in RISC-mediated silencing[27].

## MICRORNAS IN DEVELOPMENT

### *C. elegans*

### *Developmental Timing*

In *C. elegans*, miRNAs, as well as exogenous double-stranded RNAs (dsRNAs), are processed by the *C. elegans* Dicer gene, *dcr-1*[17,19,28]. Removal of Dicer, through direct mutation of the Dicer locus[17,28] or depletion by RNA interference (RNAi)[19] results in animals that are unable to generate an RNAi response and do not process miRNAs such as *lin-4* and *let-7*. Mutant worms have defects in oogenesis and are sterile, suggesting a role for miRNAs and/or RNAi in germline development[17,28]. In addition, loss of *dcr-1* results in heterochronic defects as a result of the misregulation of miRNA target genes[17,19,28].

The first two miRNAs to be discovered were identified as a result of their involvement in developmental timing in *C. elegans*. During the late L1 stage, the *lin-4* miRNA is upregulated and binds to the 3' UTR (untranslated region) of the *lin-14* gene, resulting in translational inhibition[2] and degradation of the *lin-14* transcript[25]. Another miRNA, *let-7*, was similarly found to regulate developmental timing in *C. elegans* during the L4-to-adult transition by negatively regulating *lin-14*, *lin-28*, and *lin-41* through interactions with their 3' UTRs[3]. Recently, two labs have characterized a number of additional miRNAs involved in the *C. elegans* heterochronic pathways; *mir-48*, *mir-84*, and *mir-241* were found to share a high degree of sequence similarity to *let-7* at their 5' ends. The region of similarity includes an exact, 8-nt match in the part of the miRNA that has been postulated to be essential for target site selection[29,30]. Mutational analysis revealed that these *let-7* family members have redundant functions in regulating the L2-L3 transition in *C. elegans* and may also function with *let-7* to regulate the L4-adult transition[31,32].

The 5' ends of the *let-7* family members are highly conserved and may act together to repress a common target, *hbl-1*[32]. miRNAs bind target sequences with imperfect complementarity, however, several groups have observed regions of perfect complimentarity between the 5' end of the miRNA and the 3' UTR of the target gene[33,34,35,36,37]. Recently, Lewis et al. used a bioinformatics approach to test the hypothesis that the 5' portion of the miRNA is essential for target site recognition[29,30]. Using TargetScan, a computer algorithm for predicting miRNA target genes, they found the most reliable heptamer sequence for miRNA target prediction resided at the 5' end of the miRNA (nucleotides 2–8). This observation may explain the functional redundancy reported for the *let-7* family in *C. elegans*. Variances in their 3' sequences may allow each miRNA to carry out a different function. For example, unlike *mir-48*, *mir-84*, and *mir-271*, *let-7* can target *lin-41* through significant complimentarity to the 3' end of the *let-7* miRNA[32,38]. These observations suggest that conservation at the 5' end of miRNAs may contribute to functional redundancy between related members, while sequence variation at the 3' end allows for family members to target distinct genes[33,34].

## *Neuronal Cell Fate*

A conserved feature of nervous systems is that while they develop in a morphologically symmetrical pattern, they exhibit functional asymmetry[39]. In *C. elegans*, the ability to respond to different chemosensory cues is controlled by two bilateral taste receptor neurons[40]. Although both neurons arise from a common precursor state, they acquire the ability to respond to different environmental signals by differential expression of guanyl cyclase receptor genes (gcy). To elucidate how asymmetry is established in the two neurons, ASE right (ASER) and ASE left (ASEL), Johnston and Hobert[41] conducted a genetic screen to identify neuronal asymmetry mutants. Identification of the *lsy-6* mutant resulted in an ASEL to ASER transformation and expression of the ASER-specific *gcy-5* gene in both ASE neurons. Surprisingly, cloning of the mutation revealed that *lsy-6* encoded a miRNA that is normally asymmetrically expressed only in ASEL. The *lsy-6* miRNA was found to have target sites in the homeobox gene *cog-1*, which in turn sets off a signaling cascade resulting in the differential gcy expression patterns. Therefore, in the ASEL, *lsy-6* inhibits *cog-1* and results in expression of *gcy-7*. In the ASER, there is no expression of *lsy*-6; therefore, *cog-1* is expressed, leading to expression of *gcy-5*.

Another mutant, *ot26*, also results in an ASEL to ASER transformation phenotype[42]. Cloning of this mutation identified the C2H2 zinc-finger transcription factor *die-1*. *die-1* was found to regulate expression of the *lsy-6* miRNA, thus asymmetric *die-1* expression resulted in asymmetric *lsy-6* expression. Increasing the complexity of the pathway responsible for ASE asymmetry, *die-1* was found to be the target of *mir-273*[42]. Interestingly, while *mir-273* seemed to be at the top of the signaling cascade, removal of *lsy-6* resulted in symmetrical *mir-273* expression, such that expression was derepressed in the ASEL due to activation of the *lsy-6* target, *cog-1*[43]. Therefore, asymmetric gcy expression in the ASER and ASEL neurons is regulated by a complex feedback loop involving two asymmetrically expressed miRNAs (Fig. 1). This new role for miRNAs provides insight into possible mechanisms controlling the functional asymmetry of the nervous system.

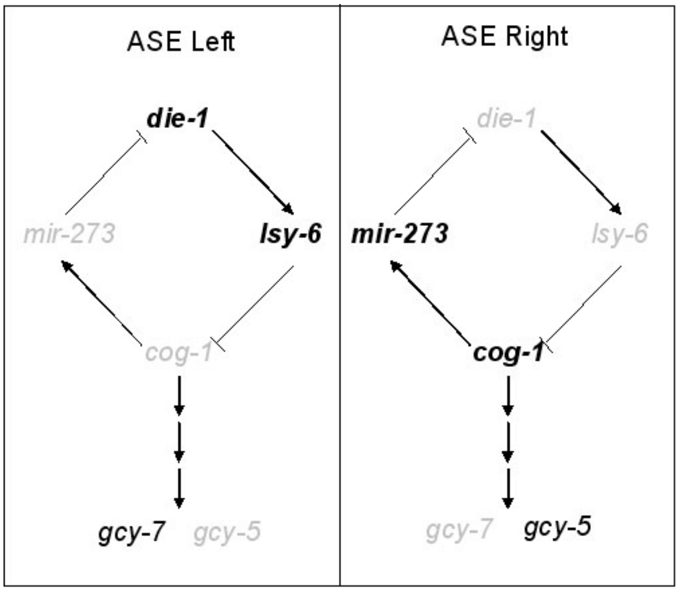

**FIGURE 1**. ASE neuronal cell fate in *C. elegans* is controlled by a feedback loop. Summary of pathway generating *gcy* asymmetry in the ASEL and ASER. See text for details. Genes listed in black represent expressed genes. Genes in grey are not expressed.

## *Drosophila*

### *Germline Stem Cell Maintenance*

Unlike *C. elegans*, zebrafish, and mammals, there are two homologs of dicer in *Drosophila*: *dicer-1* and *dicer-2*[44]. *Dicer-2* processes small interfering RNA (siRNA) precursors such as long dsRNAs, and *dicer-2* mutants are viable and fertile. *Dicer-1*, complexed with the dsRNA binding protein (dsRBP) Loquacious (*loqs*), processes endogenous miRNAs and may partially function in the siRNA pathway[45,46]. Mutations in *dicer-1* cause developmental abnormalities in the eye and surrounding sensory bristles, as well as patterning defects[44]. Similar to *C. elegans*, deletion of *dicer-1* results in germline defects. Hatfield et al.[47] observed that female *dicer-1* mutants had reduced numbers of gametes, however, no defects were apparent in germline stem cell (GSC) maintenance or survival; GSC were still present in both ovaries and testes and were able to maintain their stem cell fate. Instead, the frequency of GSC cell division was reduced at the G1/S cell cycle checkpoint in *dicer-1* null flies.

Transition through the G1/S checkpoint occurs by repression of the checkpoint inhibitor *Dacapo* (*Dap*)[48]. Interestingly, *Dap* contains multiple miRNA binding sites in its 3' UTR and a transgene lacking these sites mimics the phenotype observed on loss of *dicer-1*, suggesting that miRNAs may directly regulate the GSC cell cycle by repressing the checkpoint inhibitor *Dap*[47].

*Drosophila loqs* mutations result in the inability to process miRNAs[49]. The phenotype of *loqs* mutant flies is similar to the germline defects observed in *dicer-1* null flies[45]. In the mutant, ovaries are devoid of GSCs, however, small numbers of germ cells are present within the gonad. This suggests that GSCs must have been present at an earlier stage, and loss of miRNA processing caused GSCs to either die or differentiate into another cell type[45]. Therefore, like *C. elegans* where loss of *Dicer* also results in sterility, miRNA processing plays a crucial role in the differentiation and maintenance of the *Drosophila* germline. It will be interesting to see if miRNAs play similar roles in other stem cell populations.

### *Cell Proliferation and Cell Death*

Forward genetic screens have the advantage of identifying genes based on mutant phenotypes of interest. Using such screens, two labs identified *Drosophila* miRNAs involved in cell proliferation and apoptosis. The first group discovered the *bantam* mutation during a gain-of-function screen for genes involved in tissue overgrowth, suggesting a role for *bantam* in cell proliferation[50]. Homozygous *bantam* mutants were growth retarded and died during the early pupal stage, while heterozygotes were essentially normal. Using a novel computer program to identify *bantam* target genes, Brennecke et al. discovered *hid*, a known inducer of apoptosis[50]. *Hid* contains multiple *bantam* binding sites in its 3' UTR, suggesting that one function of *bantam* is to decrease apoptosis during embryogenesis by negatively regulating *hid*. It is unlikely that *bantam* targeting of *hid* alone is sufficient to cause the tissue overgrowth phenotype observed in the mutants, because other antiapoptotic factors, which block cell death, show no increase in cell proliferation when overexpressed. Therefore, the cell-proliferative and apoptotic-blocking functions of *bantam* may depend on additional *bantam* targets.

A second gene, *mir-14*, has also been implicated as a suppressor of cell death in *Drosophila*[51]. While there is no *in vivo* evidence, *Drice*, a proapoptotic gene, has been identified as a possible *mir-14* target based on the presence of *mir-14* binding sites in its 3' UTR. Interestingly, in addition to the observed increase in cell death in *mir-14* mutants, there is an increase in fat levels, suggesting that *mir-14* may regulate fat metabolism in addition to apoptosis[51].

*Bantam* and *mir-14* represent the first two miRNAs outside of *C. elegans* to be characterized using genetic mutations, and provide examples of how individual miRNAs can mediate various processes; *bantam* regulates genes involved in apoptosis and cell proliferation, while *mir-14* targets genes involved in apoptosis and fat metabolism. While no *bantam* homologs have yet been identified in mouse or humans, three homologs exist in *C. elegans* (*mir-80, mir-81, mir-82*)[4,6,50]; however, the genes these miRNAs target have not been identified and their potential roles in cell proliferation are unknown.

## Zebrafish

### *Brain Morphogenesis*

miRNAs are critical regulators of developmental pathways in invertebrates. High conservation of miRNAs between invertebrate and vertebrate species suggests that miRNAs may also be important for vertebrate development. Recent experiments in zebrafish and mice have shown this to be true.

In zebrafish, targeted disruption of *dicer* results in a loss of miRNA processing and developmental arrest around 8 days postfertilization[52]. This is after most organ systems have formed. It has been suggested that the presence of maternal Dicer masks the role miRNAs may play in earlier aspects of embryogenesis. Giraldez et al.[53] addressed this issue in zebrafish by using a germline replacement technique to generate wild type fish containing mutant germ cells (null for *dicer*). These chimeric fish, although lacking *dicer* activity in the germline, were fertile and, on intercrossing, produced embryos lacking both maternal and zygotic *dicer* (MZdicer). While MZdicer mutant embryos displayed normal axis formation, severe defects were observed during gastrulation, somitogenesis, and development of the heart and brain. These results suggest that, unlike *C. elegans* and *Drosophila*, Dicer is not required for germ cell development in zebrafish. Early patterning and cell specification occurred normally in MZdicer mutants, but later morphogenic processes were disrupted, suggesting that miRNAs play important roles in differentiation or maintenance of specific tissues.

The MZdicer mutant lacks all mature miRNAs. Therefore, it was not possible to characterize the roles of individual miRNAs during embryonic development. To assess what specific miRNAs might contribute to the embryonic defects observed in the MZdicer mutant, Giraldez et al.[53] used a complementation approach in which miRNAs were cloned from several early developmental stages and injected into MZdicer zebrafish in an attempt to rescue the mutant phenotypes. One family identified in this screen, the *mir-430* family, was found to be highly expressed during gastrulation and somitogenesis. To determine whether loss of *mir-430* processing caused the defects observed in the MZdicer mutant, *mir-430* duplexes were injected into mutant embryos. Interestingly, the *mir-430* duplexes rescued defects in brain development and partially rescued defects in gastrulation and somitogenesis; however, heart defects remained unchanged[53]. These results implicate the *mir-430* family in controlling specific aspects of zebrafish morphogenesis. Further investigation of miRNAs and their target genes in the MZdicer mutant will help to elucidate the mechanisms by which miRNAs control morphogenic processes such as development of the zebrafish brain.

## Mice

### *Embryonic Development*

To determine the functional importance of miRNAs in a mammalian system, several labs have generated mice deficient in Dicer. Loss of Dicer results in animals that cannot process miRNAs and the misexpression of downstream miRNA target genes[54,55,56,57]. Like *C. elegans* and zebrafish, mice contain a single dicer gene[58]. Targeted deletion of the second RNase III domain of Dicer results in embryonic lethality prior to E7.5, with a loss of expression of the stem cell marker *Oct4*[54]. Maternally contributed Dicer may allow for development prior to E7.5. The absence of development past E7.5 and lack of *Oct4* expression suggests that Dicer and miRNAs are key regulators of embryonic development and possibly of stem cell maintenance[54].

A second, independent Dicer deletion allele was recently constructed in which exons one and two were removed. Embryos homozygous for this allele produce a partially functional protein that leads to embryonic lethality between E12.5 and E14.5[57]. This mutation results in defects in blood vessel formation in yolk sacs as well as in the embryo proper. Branching vessels were observed in mutant embryos, suggesting that early stages of vessel formation were unaffected. Several genes involved in vasculogenesis and angiogenesis were misregulated in the mutants, including *flt1*, *kdr*, *vegf*, and *tie-1*. While the hypomorphic mutation results in prolonged viability, mutant embryos do not survive until birth, reinforcing the importance of miRNAs during embryonic development. Additionally, data obtained from this hypomorphic allele indicate that miRNAs play a role in the maintenance of blood vessels during embryogenesis.

## Limb Development

The early embryonic lethality of Dicer mouse mutants prevented the examination of miRNA functions at later developmental stages. Our lab, in collaboration with Mike McManus (UCSF) generated a conditional allele of *dicer*[56]. This allele allowed us to remove Dicer from a number of locations in the mouse limb using cre recombinase (see [59] for an excellent review of site-specific recombinases). In tissues from which Dicer was removed, miRNAs were not processed. Using the Dicer conditional allele in combination with various cre alleles, we were able to analyze the effects of miRNAs in the development of various mouse tissues. To date, we have removed Dicer specifically from the limb mesoderm and ectoderm in an attempt to determine the potential role for miRNAs in limb morphogenesis and patterning.

Using a limb-mesodermal prxcre transgene, conditional mice were generated that were null for *dicer* in the entire limb mesoderm[56]. This resulted in a significant decrease in limb size compared to wild type littermates. This size difference can, in part, be explained by a marked increase in cell death within the mesoderm of limbs that lack Dicer. Interestingly, skeletal preparations of mutant limbs revealed that, while strikingly smaller than normal, most components of the limb were present, although there was a reduction in the number of forelimb digits. Using a conditional allele of *dicer*, we showed that loss of miRNA processing specifically in the limb mesoderm results in proper patterning of proximal limb components, but increased apoptosis within the mesoderm and a significant loss of forelimb digits.

Two signaling centers have been identified that control patterning of the limb bud: the zone of polarizing activity (ZPA), which controls anterior-posterior patterning and digit specification, and the apical ectodermal ridge (AER), which controls proximal-distal patterning and limb outgrowth[60]. To examine whether miRNAs in either of these signaling centers control limb patterning, Dicer was specifically removed from the ZPA or the AER[56]. Removal of Dicer from the ZPA resulted in limbs containing all five digits, however, digits four and five, which are composed of cells from the ZPA, were reduced in size due to an increase in cell death. Removal of Dicer in the AER resulted in limbs that were phenotypically indistinguishable from wild type. These results suggest that miRNAs in these signaling centers do not control digit specification or limb patterning. However, while both cre transgenes turn on at approximately E9.5, Dicer protein and/or mature miRNAs may persist following cre recombination of Dicer, depending on their rate of turnover. This is consistent with preliminary data from our lab where, using Affymetrix GeneChips, changes in gene expression were not detectable until almost 2 days after transcription of functional *dicer* mRNA had ceased. Use of cre alleles that express in the precursors of cells that give rise to the ZPA and AER are needed to further analyze the role miRNAs may be playing in these structures. This is especially true for cells of the AER which exist only during a limited time in early limb development.

## Hematopoeitic Lineage Differentiation

Lymphocytes mediate the immune response through the recognition of foreign antigens by receptors on their cell surface[61]. B cells and T cells are lymphocytes that arise from a common lymphoid precursor cell, but mature in different locations in the body (B cells mature in the bone marrow, while T cells mature in the thymus). Due to the maturation of B and T cells in different locations, different sets of receptors are expressed in these two cell populations. Generating and maintaining appropriate numbers of both cell types is essential for proper immune function. Recently, miRNAs have been implicated in influencing the differentiation of lymphoid cell types.

To study the roles of miRNAs during T cell development, Cobb et al. generated a conditional allele of *dicer*[55]. Using a cre transgene specifically expressed during the earliest stages of T cell development, they were able to assess the effects of Dicer loss on T cell differentiation. In the thymus, immature T cells differentiate to express one of two T cell receptors: αβ or γδ[61]. The αβ-expressing cells compose over 95% or the T cell population and play a critical role in antigen response. The γδ cell population represents approximately 5% of the T cell population and is present in epithelial cells, where their function is not completely understood[61]. Removal of *dicer* in early T cells results in a decreased number of αβ-expressing T cells, causing an increase in the percentage of γδ-expressing cells[55]. The reduced numbers of αβ T cells may be due to increased cell death as *dicer* mutant thymocytes undergo increased apoptosis. Therefore, although loss of miRNA processing caused increased cell death specifically in αβ T cells, γδ cells were unaffected. Additionally, surviving αβ cells could further differentiate into cytotoxic and helper T lymphocytes, suggesting that miRNAs play a limited role in controlling T cell lineage choice.

To identify miRNAs important for hematopoeitic cell differentiation, several groups have cloned miRNAs from the bone marrow, thymus, and spleen. miRNAs were found to be expressed differentially at various stages of hematopoeitic differentiation[8,62,63]. One miRNA, *mir-181*, was upregulated as the lymphoid precursor cell differentiates into B cells[62]. To assess the function of this miRNA on B cell development, *mir-181* was ectopically expressed in the hematopoietic progenitor cells. This resulted in a twofold increase of B cells with no increase in T cells. Conversely, two other miRNAs identified in their screen had the opposite result; overexpression of *mir-14*2 or *mir-223* caused a 30–40% increase in T lineage cells with no effect on B lineage cells. Chen et al.[62] further tested their results *in vivo*. Irradiated mice were reconstituted with virally infected lymphoid progenitor cells overexpressing *mir-181*. In these mice, B cell numbers increased twofold over controls, while T cell numbers dropped 88%. These results strongly implicate *mir-181* in controlling B cell lineage choice and suggest that other miRNAs, such as *mir-14*2 and *mir-223*, may also be involved in lymphoid cell differentiation.

## Neural Cells

Some miRNAs are expressed in highly tissue-specific patterns, suggesting that they may function in cell-type-specific differentiation. Several groups have shown that miRNAs are highly enriched in the brain and that many of these miRNAs are expressed in spatially and temporally restricted patterns[64,65,66]. For example, both neurons and astrocytes (a subtype of glial cell that supports neurons) arise from a common precursor stem cell[67]. Neurons, however, are enriched for *mir-124a* and *mir-128*, while astrocytes express *mir-23*, *mir-66*, and *mir-69*[67]; *mir-124a* is highly expressed in the brain[65,66]. Interestingly, overexpression of *mir-124a* in HeLa cells results in downregulation of over 170 genes[68]. The dramatic change in gene expression caused by overexpression of a single miRNA highlights how individual miRNAs may function to regulate large numbers of target genes. In HeLa cells, *mir-124a* overexpression led to decreased expression of genes normally expressed at very low levels in the brain. The resulting HeLa cells acquired gene expression profiles that resembled those of mature neurons. Therefore, rather than promoting differentiation, miRNAs in the brain may function to silence non-neuronal genes and help to maintain the neuronal cell state.

## Insulin Secretion

Pancreatic islet cells, made up of glucagon-secreting α cells and insulin-secreting β cells, maintain the proper balance of blood glucose levels[69]. Increased uptake of glucose by β cells leads to the secretion of insulin-containing vesicles by exocytosis. Recent data suggest that an islet-specific miRNA may contribute to insulin secretion by β cells[70].

To explore the roles of miRNAs in regulating glucose metabolism, Poy et al.[70] cloned miRNAs from the glucose-responsive pancreatic β cell line MIN6 and the α cell line TC-1. From this screen, *mir-375* was identified as being an islet-specific miRNA. To determine if this novel miRNA was involved in glucose metabolism, *mir-375* expression was downregulated in MIN6 cells using siRNAs. Surprisingly, following glucose stimulation, MIN6 cells with decreased *mir-375* had increased insulin secretion compared to control cells. Conversely, *mir-375* overexpression resulted in decreased insulin secretion. Further analysis revealed that while no defect was observed in insulin synthesis or in intracellular $Ca^{2+}$ signaling (essential for exocytosis of secretory vesicles[71]), there was a block in exocytosis on overexpression of *mir-375*. Using a computer algorithm to search for miRNA target genes that might be involved in the exocytosis defect, *myotrophin* (*Mtpn*) was identified as a *mir-375* target[70]. Decreased expression of Mtpn in MIN6 cells resulted in reduced insulin secretion, mimicking *mir-375* overexpression. Therefore, in MIN6 cells, *mir-375* can effect insulin secretion by blocking exocytosis of insulin-containing vesicles, possibly by repressing its target gene, *Mtpn*. The identification of an islet-specific miRNA involved in insulin secretion adds new complexity to the β cell pathway regulating glucose metabolism and provides additional therapeutic targets for metabolic disorders such as diabetes.

## Genomic Imprinting

Mammalian development requires the proper inheritance of a maternal and paternal genome[72]. Embryos inheriting two maternal (gynogenetic) or two paternal genomes (androgenetic) die during early stages of development[72]. The nonequivalence of the maternal and paternal genomes can be attributed to the monoallelic expression of specific genes based on their “parent of origin”, a phenomenon known as genomic imprinting[73]. Recently, several miRNAs were identified in a region subject to genomic imprinting, the *Dlk1-Gtl2* imprinted locus[74,75]. Within this locus, the protein-coding genes *Dlk1*, *Dio3*, and *Peg11* (*Rtl1*) are expressed exclusively from the paternal chromosome, while several noncoding RNA transcripts, *Gtl2*, *Mirg*, and anti-*Peg11*, are expressed maternally[76]. Seitz et al.[75] identified several miRNAs transcribed within the maternally expressed anti-*Peg11* and *Mirg* noncoding transcripts. Interestingly, *mir-136* and *mir-127*, encoded within the anti-*Peg11* transcript, exhibit perfect complimentarity with *Peg-11*. Cloning and sequencing of *Peg11* cleavage products corresponding to the anti-*Peg11*–encoded miRNAs confirmed that the imprinted miRNAs regulated *Peg11* transcription by RISC-mediated cleavage of the transcript[75].

The *Dlk1-Gtl2* imprinted region includes the callipyge (CLPG) locus[77]. Originally identified in sheep, the callipyge mutation leads to muscle overgrowth, possibly resulting from increased expression of the *Dlk1* and *Peg11* genes[78]. However, the mutation only results in a phenotype in the heterozygous state when paternally inherited. A recent publication provides a new explanation for the complex inheritance of the callipyge phenotype[74]. When the mutation is inherited maternally, increased levels of anti-*Peg11*–encoded miRNAs are observed. Conversely, paternal inheritance results in increased *Peg11* expression, resulting in the muscle hypertrophy phenotype. When the mutation is homozygous, increased levels of miRNAs, derived from the anti-*Peg11* transcript, target the overexpressed *Peg11* transcript for degradation. This results in decreased *Peg11* transcript, increased *Peg11* cleavage products and no hypertrophic phenotype. Therefore, miRNAs in this imprinted locus act *in trans* to maintain the proper transcript levels of their target gene, *Peg11* (Fig. 2).

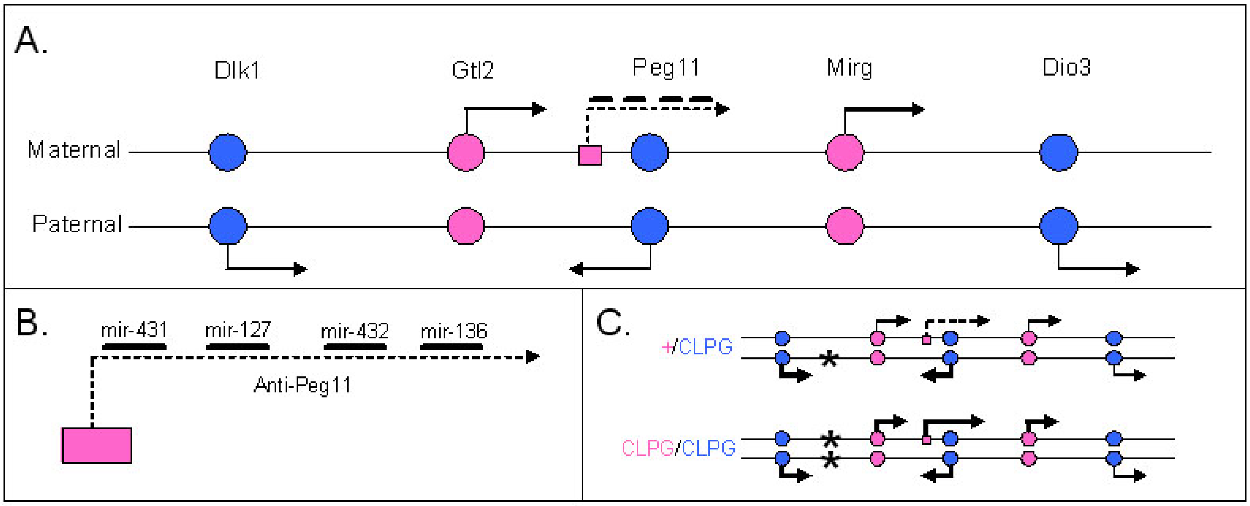

**FIGURE 2**. MiRNA expression in the *Dlk1-Gtl2* imprinted locus. (A) Schematic representation of the maternal and paternal alleles of the *Dlk1-Gtl2* imprinted locus. (B) An enlarged view of the *anti-Peg11* transcript including several miRNAs shown to be overexpressed when inherited with the CLPG mutation. (C) The effect of the CLPG mutation on gene expression in the *Dlk1-Gtl2* locus. Only wildtype/CLPG (+/CLPG) and CLPG/CLPG are shown. See text for details. Blue circles represent paternally expressed genes. Pink circles represent maternally expressed genes. The maternally expressed *anti-Peg11* transcript is depicted as a pink square and a dotted line. Genes that are transcribed are indicated with an arrow. Genes that are overexpressed are depicted by bold arrows. An asterisk (*) marks the CLPG mutation.

The identification of imprinted miRNAs in the *Dlk1-Peg11* locus provides an explanation for the complex inheritance of the callipyge phenotype and provides a new mechanism for a trans-acting, imprinted, noncoding RNA. As noncoding RNAs are a common theme for imprinted loci, it is possible that other imprinted, noncoding RNA transcripts may share similar regulatory roles.

## Muscle

While certain miRNAs are broadly expressed, others are expressed at specific developmental stages, in a cell-type specific manner. *mir-1* has been observed by several groups to be highly expressed in the heart and muscle[65,79,80]. Based on its expression pattern, *mir-1* was suspected to play an important role in muscle cell differentiation, however, studies in mice and *Drosophila* have revealed divergent roles for this highly conserved miRNA.

In the mouse, the transcription factor Hand2 maintains the proliferative population of undifferentiated myocytes[81,82]. Myocyte differentiation, mediated by the transcription factor Serum Response Factor (SRF)[83], is accompanied by downregulation of Hand2, which results in cessation of mitotic cell divisions[81,82]. Zhao et al.[79] recently found that the two *mir-1* genes (*mir-1-1* and *mir-1-2*) were expressed in the developing heart and that their expression was dependent on SRF binding sites in their promoters. Expression of both *mir-1* genes in the somites was dependent on the transcription factors MyoD and Mef2. To understand the role of these miRNAs in cardiomyocyte development, *mir-1* was overexpressed in the developing heart. Overexpression of the miRNA resulted in decreased proliferation of cardiomyocytes, heart failure, and lethality by E13.5. Using a new algorithm for identifying miRNA targets, Zhao et al. identified and validated *Hand2* as a target for *mir-1*. Therefore, in the mouse heart, SRF activates *mir-1* which then causes repression of *Hand2,* allowing cardiomyocytes to differentiate.

In *Drosophila, mir-1* is expressed in mesodermal cells early in development, and like the mouse, the later restriction of *mir-1* to heart and muscles requires the activity of *mef2*[80]. However, a null mutation of *mir-1* had no detectable effect on heart or muscle as both tissue types were morphologically and functionally normal. Later in development, *Drosophila* larvae could not transition from the first instar to second instar stage, larvae became increasingly lethargic and eventually died from severe disruption of the body wall musculature[80]. The transition from the first to second instar stage is marked by a ~200-fold increase in body mass over 4 days, however, the increased muscle growth is due to expansion of cell size by endocytic DNA replication (replication without cell division) and is not due to increased cell proliferation[84]. Therefore, in *Drosophila*, *mir-1* likely is necessary for maintenance of muscle integrity or muscle cell identity. Consistent with this hypothesis, human *mir-1* has been shown to restrict expression of nonmuscle genes in muscle cells[68].

The results in *Drosophila* seem surprising when considering the severe effect of overexpressing mouse *mir-1* in the heart and with *mir-1* expression being specifically expressed in the developing heart of both species. It seems that loss of *mir-1* in *Drosophila* does not effect cardiomyocyte differentiation, or the effect is minimal, and a functional heart can still form. Although *mir-1* overexpression in the mouse has significant effects on expression of its downstream target *Hand2*, and severe morphological defects, it will be of interest to see if loss of *mir-1* also causes an early embryonic heart defect or if the phenotype will resemble that in *Drosophila*.

**TABLE 1**
**miRNA Functions in *C. elegans, Drosophila*, Zebrafish, and Mouse**

| Organism | miRNA(s) | miRNA Function | References |
|---|---|---|---|
| *C. elegans* | lin-4, let-7, mir-48, mir-84, mir-241 | Developmental timing | [2,3,17,19,28,31,32] |
| | Loss of dicer | Oogenesis | [17,28] |
| | lsy-6, mir-273 | Asymmetric neuronal cell fate | [41,42,43] |
| *Drosophila* | Loss of dicer | GSC maintenance | [47] |
| | bantam, mir-14 | Cell proliferation, cell death, fat metabolism | [50,51] |
| | mir-1 | Muscle cell maintenance or integrity | [80] |
| Zebrafish | mir-430 family | Brain morphogenesis | [53] |
| Mouse | Conditional loss of dicer | Blood vessel maintenance | [57] |
| | Conditional loss of dicer | Limb development | [56] |
| | Conditional loss of dicer | T lymphocyte development | [55] |
| | mir-181 | B lymphocyte development | [62] |
| | mir-375 | Insulin secretion | [70] |
| | mir-431, mir-127, mir-432, mir-136 | Gene regulation in an imprinted locus | [74,75,85] |
| | mir-1 | Heart and muscle development | [79] |

## CONCLUSIONS

Since the discovery and identification of hundreds of miRNAs in both invertebrate and vertebrate species, the functional importance of this new class of noncoding RNA is slowly being realized. miRNAs are now known to carry out diverse functions and even highly conserved miRNAs may play different developmental roles in different species. The analysis of organisms carrying mutations in Dicer has been a powerful approach for studying the roles of all miRNAs during development. The recent development of conditional Dicer alleles in mice will further our understanding of miRNA functions in specific tissues. However, uncovering the roles of individual miRNAs remains challenging due to possible functional redundancy between miRNAs. This problem is highlighted in the mouse model system in which it is expensive and time consuming to create multiple knockout lines. Deletion mutants will nevertheless provide valuable information on miRNA functions and help us gain a better understanding of the roles they play during development.
